# Density‐dependent patterns of multivariate selection on sperm motility and morphology in a broadcast spawning mussel

**DOI:** 10.1002/ece3.8514

**Published:** 2022-02-09

**Authors:** Jessica H. Hadlow, Rowan A. Lymbery, Jonathan P. Evans

**Affiliations:** ^1^ Centre for Evolutionary Biology School of Biological Sciences The University of Western Australia Crawley WA Australia

**Keywords:** broadcast spawner, gamete interactions, gamete limitation, sexual selection, sperm competition

## Abstract

Sperm cells exhibit extraordinary phenotypic variation, both among taxa and within individual species, yet our understanding of the adaptive value of sperm trait variation across multiple contexts is incomplete. For species without the opportunity to choose mating partners, such as sessile broadcast spawning invertebrates, fertilization depends on gamete interactions, which in turn can be strongly influenced by local environmental conditions that alter the concentration of sperm and eggs. However, the way in which such environmental factors impact phenotypic selection on functional gamete traits remains unclear in most systems. Here, we analyze patterns of linear and nonlinear multivariate selection under experimentally altered local sperm densities (densities within the capture zone of eggs) on a range of functionally important sperm traits in the broadcast spawning marine mussel, *Mytilus galloprovincialis*. Specifically, we assay components of sperm motility and morphology across two fertilization environments that simulate either sperm limitation (when there are too few sperm to fertilize all available eggs), or sperm saturation (when there are many more sperm than required for fertilization, and the risk of polyspermy and embryonic failure is heightened). Our findings reveal that the strength, form, and targets of selection on sperm depend on the prevailing fertilization environment. In particular, our analyses revealed multiple significant axes of nonlinear selection on sperm motility traits under sperm limitation, but only significant negative directional selection on flagellum length under sperm saturation. These findings highlight the importance of local sperm densities in driving the adaptation of sperm phenotypes, particularly those related to sperm motility, in broadcast spawning invertebrates.

## Graphical abstract

For externally fertilizing species, such as sessile broadcast spawning invertebrates, fertilization depends on gamete interactions, which are strongly influenced by environmental conditions that alter gamete concentrations. We analyze patterns of multivariate selection under experimentally altered local sperm densities on a range of functionally important sperm traits in a model broadcast spawning mussel. Our findings highlight the importance of limited local sperm densities in driving the adaptation of sperm traits, particularly sperm motility, in species where gamete interactions are governed by highly variable external conditions.

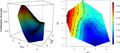

## 1 INTRODUCTION

Sperm cells are renowned for their phenotypic diversity and are among the most rapidly evolving metazoan cell types (Birkhead et al., 2009; Lüpold & Pitnick, 2018). This diversity has been broadly attributed to processes of post‐ejaculatory sexual selection such as sperm competition (Parker, 1970, 2020), but also to variation in the fertilization environment (Fitzpatrick & Lüpold, 2014; Franzén, 1956; Lüpold & Pitnick, 2018). Internal fertilizers commonly possess sperm that are morphologically complex because they need to navigate intricate female reproductive tracts and/or survive prolonged sperm storage, often in the presence of ejaculates from rival males (Fitzpatrick & Lüpold, 2014; Lüpold & Pitnick, 2018). In contrast, external fertilizers typically possess sperm that are shorter and simpler in form than those of internal fertilizers (Lüpold & Pitnick, 2018). However, external environments can nevertheless be highly heterogeneous and unpredictable, and there is growing recognition that sperm of external fertilizers can exhibit substantial variation in non‐morphological traits such as sperm velocity and swimming trajectory (Evans & Lymbery, 2020). Despite increasing interest in how environmental variation shapes selection on sperm, and recent recommendations to assess selective pressures on sperm beyond post‐ejaculatory sexual selection (Evans & Lymbery, 2020; Liao et al., 2018), there have been remarkably few studies that have explored how multivariate sperm phenotypes predict reproductive fitness under contrasting environments (Chirgwin et al., 2020; Evans & Garcia‐Gonzalez, 2016; Johnson et al., 2013; Monro & Marshall, 2016).

Broadcast spawning invertebrates—external fertilizers in which both sexes release their gametes directly into the water column—retain a reproductive strategy that is ancestral to copulation, and a considerable body of theory suggests that the selective forces acting on broadcast spawner gametes were pivotal drivers in the evolution of animal mating systems (Beekman et al., 2016; Evans & Sherman, 2013; Parker, 2014; Parker & Pizzari, 2015). In broadcast spawners, mating, and fertilization success is determined by gamete interactions, with little to no opportunity for adults to control their mating partners (Evans & Lymbery, 2020). Additionally, gamete interactions and fertilization success for broadcast spawners are impacted by the density and sex ratio of individuals participating in a spawning event (Levitan, 2004, 2005; Levitan & Ferrell, 2006; Pennington, 1985; Yund, 2000), water turbulence and flow (Crimaldi, 2012; Denny & Shibata, 1989; Levitan, 2018), and the timing and rate of gamete release (Benzie & Dixon, 1994; Marshall & Bolton, 2007; Olito & Marshall, 2019). As a result, local sperm densities (i.e., within the “capture zone” of individual ova; Levitan, 2018) range from conditions that result in sperm limitation, when there are too few sperm to fertilize all available eggs, to sperm saturation, where an excess of sperm heightens the risk of polyspermy (multiple sperm fertilizing a single egg), leading to embryonic failure (Levitan, 1998; Styan, 1998; Yund, 2000). Sperm saturation often coincides with competition among ejaculates of rival males (i.e., sperm competition; Parker, 2020), although high sperm concentrations from a single male within the capture zone of a female's eggs can also occur. Such monogamous fertilization events may occur, for example, when conditions are less turbulent, resulting in less sperm mixing and therefore fewer competitive fertilizations (Levitan, 2018). Furthermore, even turbulent conditions may result in spatial distributions of gametes that lead to high local concentrations and episodic events of intense fertilization (Crimaldi & Zimmer, 2014).

Due to the unpredictability of sperm mixing, local sperm densities and the frequency of competitive or monogamous fertilizations can vary within and between spawning events, which can then generate spatially and temporally variable patterns of selection on gamete phenotypes (Evans & Lymbery, 2020; Johnson et al., 2013; Levitan, 1998). Two studies have addressed this idea with a focus on sperm morphological variation, and found that selection favors different sized sperm at different sperm densities (Johnson et al., 2013; Monro & Marshall, 2016). However, analyses of context‐dependent selection on sperm phenotypes have yet to be extended to include characteristics beyond sperm size, despite evidence that traits such as motility and velocity are critical determinants of fertilization in many species (Fitzpatrick & Lüpold, 2014) and might play important roles in fertilization dynamics under different gamete densities (Crean & Marshall, 2008). Indeed, sperm limitation has been shown to select for slower swimming, longer lived sperm that are able to search for eggs over a greater period of time (Fitzpatrick et al., 2012). In contrast, little is known about how selection targets sperm phenotypes when sperm are saturating. If sperm from multiple males are present at saturated levels, we might expect faster swimming sperm to be selected as the pressure to compete for eggs is high and outweighs the risk of polyspermic fertilizations and cell death—conditions that lead to sexual conflict over fertilization (Levitan, 2004). However, if sperm saturating conditions occur during monogamous fertilizations, as is likely to happen if a male and female in close spatial proximity are spawning simultaneously under low turbulence, then selection may favor sperm that are less competent at fertilization (e.g., less motile sperm) because high collision rates will result in polyspermy (Evans & Lymbery, 2020; Levitan, 2018).

The broadcast spawning marine mussel, *Mytilus galloprovincialis*, provides an ideal system for studying multivariate selection on sperm across multiple environments. Although previous work on this species has reported significant patterns of multivariate nonlinear selection on sperm motility and morphology in both non‐competitive (Fitzpatrick et al., 2012; Hadlow et al., 2020) and competitive fertilizations (Lymbery et al., 2018), we have yet to determine how these patterns vary when sperm concentrations change from limiting to saturating. This is highly relevant because *M. galloprovincialis* form aggregations that vary greatly in density in intertidal zones, where, as with other broadcast spawning species, local gamete densities are highly likely to change during and between spawning events.

Here, we manipulate local sperm density during staged fertilization events and analyze patterns of multivariate selection on sperm motility and morphology in *M. galloprovincialis*. Specifically, we compare patterns of selection when the number of sperm limits fertilization rates (hereafter “sperm limitation”) and when sperm are saturating (“sperm saturation”) under passive flow conditions. The mixing and aggregation of broadcast spawning gametes are thought to be initially controlled by physics at a large spatial scale, with biological processes such as sperm swimming becoming more important at the smallest scales (Crimaldi & Zimmer, 2014). Therefore, our fertilization assays are representative of fertilization after gametes have been dispersed by physical processes and when gamete biology begins to dominate fertilization outcomes. Our measure of fitness was based on the number of normal, monospermic fertilizations achieved (as polyspermic fertilizations will lead to developmental failure; Styan, 1998; see Methods). We focused on non‐competitive fertilizations for these trials in order to avoid confounding the effects of competition with density. Though sperm competition is likely to be common in aggregations of *M. galloprovincialis*, non‐competitive fertilizations may also occur as patterns of water movement and differences in the timing of spawning among conspecific males mean that high concentrations of sperm from a single male may encounter a clutch of eggs in the absence of rivals (Levitan, 2018). Our work reveals clear differences in patterns of selection on sperm traits in the two environments, a finding that has important implications for understanding the adaptive value and drivers of sperm trait variation outside of sperm competition.

## 2 MATERIALS AND METHODS

### 2.1 Study species and gamete collection

*M. galloprovincialis* is a gonochoristic, sessile marine mollusk that inhabits subpolar and temperate regions of the Northern and Southern hemispheres, including Australia's southern coastline (Daguin & Borsa, 2000; Westfall & Gardner, 2010). Individuals live in dense aggregations and reproduce by releasing gametes directly into the water column during synchronized spawning events. We collected adult *M. galloprovincialis* from Woodman Point, Western Australia (32°14′03.6″S, 115°76′25″E) in the 2019 reproductive season (June–September in Western Australia). Mussels were placed in a large tub of filtered seawater (FSW) that was heated to 26°C to induce spawning (Evans et al., 2012; Fitzpatrick et al., 2012; Lymbery et al., 2018).

When a mussel spawned, it was sexed and immediately rinsed to reduce potential contamination from gametes in the tub. Washed mussels were placed in separate 250 ml plastic cups containing ~25 ml of FSW. When gamete densities were suitably high, adults were removed and gamete concentrations were estimated. We estimated egg concentrations by counting the number of unfertilized eggs in a 5 µl homogenized subsample. To estimate sperm concentrations, we used a Neubauer hemocytometer and aliquots of ejaculates fixed in 1% buffered formalin (used to ensure that sperm were immotile during counts). These estimates were used to adjust gametes to the required concentrations (see below).

### 2.2 Experimental design

We compared selection on sperm morphology and motility traits when fertilization occurred in two different environmental conditions: sperm limitation and sperm saturation. Under natural conditions, spawned ejaculates will become diluted in the water column, with the extent of dilution largely a function of the environment (Crimaldi & Zimmer, 2014; Levitan & Petersen, 1995). Depending on conditions, an ejaculate may enter an egg capture zone after either complete or partial dilution. We therefore estimated male fitness by experimentally manipulating sperm density independent of initial ejaculate size.

Our study design incorporated 22 experimentally feasible “blocks”. Within each block, we assayed six to 12 males individually and used four to six females as egg donors. The use of pooled eggs within each block to assay male reproductive fitness ensured that we were able to control for stochastic variation in egg quality among females and male‐by‐female interaction effects at fertilization, which are known to occur in *M. galloprovincialis* (Evans et al., 2012; Lymbery et al., 2020; Oliver & Evans, 2014). Each male was haphazardly assigned to one of the two treatments (i.e., three to six males per treatment within a block). We assayed each individual male's fertilization success using pooled eggs from the females of the same block, and used this measure as our estimate of reproductive fitness (Fitzpatrick et al., 2012; Hadlow et al., 2020; Lymbery et al., 2018).

We used measures of sperm motility and morphology as phenotypic predictors of male fertilization success. For the sperm motility assays (described below), we measured sperm behavior in the presence of “egg water” (seawater containing chemicals released by unfertilized eggs) to more closely replicate the environment sperm experience while attempting to fertilize eggs (Hadlow et al., 2020). For the sperm morphological traits, we preserved subsamples of each male's ejaculate in 1% buffered formalin for later measurement. In the final analyses we only included males for which we successfully measured both sperm morphology and motility traits, yielding a total sample size of *n* = 180 males, with *n* = 90 in the sperm limitation treatment and *n* = 90 in the sperm saturation treatment.

### 2.3 Fertilization trials

Eggs from each female within a block were used to create an egg pool at a concentration of 2.0 × 10^4^ eggs ml^−1^ for use in fertilization assays. Prior to fertilization trials, this egg pool was left to sit for 1 h to allow eggs to release chemoattractants, which were required for the motility trials (see “Sperm motility”). After that time, 2 ml of the egg pool was pipetted into separate petri dishes (one dish per male), and the leftover egg pool was set aside. We added 2 ml of ejaculate from each male to separate petri dishes with the 2 ml egg aliquots. Ejaculate concentrations were standardized at 5.0 × 10^4^ sperm mL^−1^ in the sperm limitation treatment and 5.0 × 10^5^ sperm ml^−1^ in the sperm saturation treatment. After 1.5 h, aliquots from each dish were fixed in 1% buffered formalin. We later estimated male fertilization rates by scoring the number of fertilized eggs with polar bodies or undergoing normal cleavage out of 200 haphazardly sampled eggs. These ratios produced variation in fertilization success among males and did not produce ceiling (100%) or floor (0%) success rates. As anticipated, the ratio used in the sperm saturation treatment produced some abnormal fertilizations (i.e., asymmetrical cell division), which were taken as a proxy for polyspermy and therefore deemed “unsuccessful” (Levitan & Ferrell, 2006; Okamoto, 2016; Styan, 1998).

### 2.4 Sperm motility

After commencing fertilization trials, the unused pool of eggs was filtered through a 30‐µm mesh, and the filtrate (egg water) was retained for sperm motility trials using computer‐assisted sperm analysis (CASA; CEROS II, Hamilton‐Thorne). For these assays we combined 3 µl of ejaculate at 3.0 × 10^6^ sperm mL^−1^ with 3 µl of egg water (giving a final sperm concentration of 1.5 × 10^6^ sperm ml^−1^) and added this to an individual well on a 12‐well multi‐test slide, which had been previously rinsed in 1% polyvinyl alcohol to prevent sperm sticking to the slide (Fitzpatrick et al., 2012; Hadlow et al., 2020; Lymbery et al., 2018). Sperm motility was analyzed immediately. This concentration of sperm allowed us to track sperm motility from an individual male within a reasonable timeframe while still ensuring that CASA can distinguish between individual sperm cells, and is below the recommended maximum concentration for CASA (Lu et al., 2014). We used the same sperm concentration for males in both treatments because motility parameters are not affected by sperm concentration unless that concentration is greater than recommended (Lu et al., 2014). We tracked an average (±SEM) of 109 ± 0.51 motile sperm per male. Previous work has reported high within‐sample repeatability for these CASA measures in *M. galloprovincialis* using the exact methods employed in the present study (Fitzpatrick et al., 2012).

The CASA generates several motility parameters describing sperm velocity, linearity, and cell head movements. From these, we selected parameters representing distinct traits, which were not significantly colinear (see Section 3), and have previously been shown to predict fertilization success in *M. galloprovincialis* (Hadlow et al., 2020; Lymbery et al., 2018). These traits were beat‐cross frequency (BCF), path linearity (LIN), the percentage of motile sperm (PM), and curvilinear velocity (VCL). Threshold values for static cells were set at 4 μm s^−1^ for straight‐line velocity and 19.9 μm s^−1^ for average path velocity (Fitzpatrick et al., 2012; Hadlow et al., 2020; Lymbery et al., 2018).

### 2.5 Sperm morphology

We preserved 450 μl of each male's ejaculate in 1% buffered formalin. Subsamples were taken directly from individual spawning cups and were stored at room temperature (approx. 22°C). We photographed 20 sperm from each male using an Olympus BX41 microscope (Olympus) and an EOS 600D camera (Canon) at 800× magnification. We measured sperm head length and flagellum length using ImageJ version 1.48 (Collins, 2007).

### 2.6 Selection analyses

All analyses were conducted using R version 3.6.0 (R Development Core Team, 2019). Before formally estimating selection gradients for sperm traits, we assessed whether linear and nonlinear (quadratic and correlational) relationships between the traits and fitness varied between the treatments (Chenoweth & Blows, 2005). We standardized all phenotypic traits to a mean of 0 and standard deviation of 1 prior to any analyses (Lande & Arnold, 1983). We then used a sequential model‐building approach and log‐likelihood ratio tests to compare the fit of generalized linear models (using the package “glmmTMB”; Brooks et al., 2017) with and without trait‐by‐treatment interactions, and with a random effect of block ID (Chenoweth & Blows, 2005; Chenoweth et al., 2012; Draper & John, 1988). Block ID was included as a random effect in these models to account for the repeated use of egg pools within each block (i.e., one egg pool per block). A likelihood ratio test revealed significant among‐block variation in fertilization success within each treatment (sperm limitation: LRT, *χ* ^2^ = 2184.7, df = 1, *p* < .001; sperm saturation: LRT, *χ* ^2^ = 1354.5, df = 1, *p* < .001). We therefore retained block ID as a random term in these models, but also confirmed with supplementary analyses that no single block significantly changed the fit of our models when each block was successively omitted from the dataset (Table A1). The sequential model‐building procedure began with a reduced model including fixed effects of treatment and linear trait terms and the proportion of successfully fertilized eggs as the response variable, which we then compared to a model including interactions between linear terms and treatment. We repeated this process (i.e., comparing models with and without trait‐by‐treatment interactions) for models that included either linear and quadratic terms, or linear, quadratic, and correlational terms. Betabinomial distributions and logit link functions were used to account for overdispersion in these models (dispersion parameters ranged from 7.75 to 13.6). As we found evidence that the inclusion of treatment terms improved the fit of linear and nonlinear models (see Section 3), we proceeded with formal selection analyses to identify specific differences in patterns of selection occurring in the two treatments.

We used a modified approach of the multiple regression methods of Lande and Arnold (1983) to estimate linear (*β_i_*) and nonlinear (*γ_ij_*) selection gradients as the first and second partial derivatives of absolute fitness with respect to the multivariate phenotype (Morrissey & Sakrejda, 2013). These gradients were estimated separately within the two treatments. We began by fitting generalized additive models with quasibinomial error distributions (dispersion parameters ranged from 13.0 to 29.4) and logit link functions, including the proportion of fertilized eggs as the response variable, six sperm traits (BCF, LIN, VCL, PM, HL, and FL) as predictors, and a random effect of block ID, using the package “mgvc” (Wood, 2017). We then estimated the *β_i_* and *γ_ij_* gradients using the “gsg” package, and used case bootstrapping to calculate standard errors and conduct hypothesis tests for individual gradients (Morrissey & Sakrejda, 2013). Correlation between linear and quadratic terms can lead to inaccurate estimations of *β_i_* (Lande & Arnold, 1983), so we used models with only linear terms to calculate *β_i_* gradients. All first and second‐order terms were included in the models used to calculate *γ_ij_* estimates and the **γ** matrix (which contains quadratic selection gradients on the diagonal and pairwise correlational gradients off the diagonal). Models were fit separately for each treatment.

Multiple regression typically underestimates patterns of nonlinear selection (Blows & Brooks, 2003) because selection often targets combinations of more than two traits (Lande & Arnold, 1983; Phillips & Arnold, 1989). Consequently, we performed a canonical rotation of the **γ** matrix to identify major axes of the nonlinear selection surface and selection on sperm trait combinations (Phillips & Arnold, 1989). This analysis finds the eigenvectors (***m*** *_i_*) and associated eigenvalues (*λ_i_*) of **y** by eliminating pairwise correlational terms and producing a matrix, **M**, of the eigenvectors. These eigenvectors define major canonical axes of the selection surface and are loaded by combinations of the original traits. The absolute value of *λ_i_* describes the strength of nonlinear selection along each canonical axis. Positive and negative *λ_i_* correspond to concave or convex selection, respectively. We determined the overall slope of selection (*θ_i_*) along the canonical axes by rotating the original *β_i_* onto the new trait space (Blows & Brooks, 2003; Phillips & Arnold, 1989). To assess the significance of *λ_i_* and *θ_i_* we used a permutation procedure that generates null distributions for each selection gradient (Chenoweth et al., 2012; Reynolds et al., 2010). We randomly permuted fertilization success 1000 times, fit a second‐order GAM with rotated trait scores and permuted fitness, and extracted selection gradients using “gsg” to generate null distributions. We kept the canonical rotation constant for each permutation because we were interested in selection along the eigenvectors of the original **y** matrix (Chenoweth et al., 2012). For visualization of the overall selection surfaces, we fit nonparametric thin‐plate splines using the “fields” package to visualize selection on multiple axes in multivariate space (Nychka et al., 2017). Smoothing parameters were set to minimize generalized cross‐validation scores (Craven & Wahba, 1978).

We used a geometric approach to quantitatively compare selection gradients in the two treatments. We first compared the direction of linear selection in the treatments by calculating the angle between β gradient vectors from the two treatments (an angle of 0° indicates the same direction, 90° indicates orthogonal vectors, and 180° indicates opposite directions). To determine the similarity between the **y** matrices from each treatment, we compared the orientation of matrix subspaces using the Krzanowski (1979) method. This analysis compares a subset (*k*) of eigenvectors from two matrices (*k* ≤ *n*/2; *n* = number of eigenvectors in a matrix) and produces a metric bounded by 0 and *k*. A score of 0 indicates complete dissimilarity, and a score of *k* indicates perfect alignment of the subspaces. We used the three eigenvectors with the largest eigenvalues (**m1**, **m5** and **m6** in both treatments; see Section 3), which produced a metric bounded by 0 and 3. We also calculated the correlation of individual *β_i_* gradients, and of individual *γ_ij_* gradients, between treatments following Berson and Simmons (2018).

### 2.7 Phenotypic correlation analysis

We calculated pairwise full and partial correlation coefficients for sperm traits (the latter represent pairwise correlations holding all other traits constant), and used the package “ppcor” to assess the significance of partial correlation coefficients (Kim, 2015). To verify that phenotypic trait correlations were similar for the sets of males used in each treatment, we compared phenotypic correlation matrices, and partial phenotypic correlation matrices, between the two groups of males using the “MantelCor” function in the package “evolqg” (Melo et al., 2016). This function calculates Pearson correlation coefficients between corresponding elements of the matrices being compared. Significance is determined by generating a null distribution through permutation of rows and columns in one matrix, and then repeating element‐by‐element correlations. The correlation between matrices will range between −1 (matrices have opposite structures) and 1 (matrices have the same structure). We also calculated variance inflation factors (VIFs) for each phenotypic trait, which can provide information about the reliability of partial regression coefficients (see Morrissey & Ruxton, 2018).

## 3 RESULTS

### 3.1 Fertilization rates

Males in the sperm limitation treatment achieved an average of 76 ± 4 successful fertilizations (mean ± SE) out of 200, whereas males in the sperm saturation treatment achieved 100 ± 4 successful fertilizations (mean ± SE) out of 200. Of the unsuccessful fertilizations in the sperm saturation treatment, an average of 50 ± 4 (mean ± SE) were abnormal (likely polyspermic) fertilizations.

### 3.2 Linear and nonlinear selection on individual traits

The sequential model‐building analysis revealed that linear (LRT, *χ* ^2^ = 39.0, df = 6, *p* < .001) and correlational (LRT, *χ* ^2^ = 30.0, df = 15, *p* = .012) selection on sperm traits differed significantly between the two treatments. In contrast, quadratic selection on traits did not significantly differ between the treatments (LRT, *χ* ^2^ = 1.1, df = 6, *p* = .982).

Formal selection analysis revealed significant negative linear selection on head length, significant positive linear selection on flagellum length, and marginally non‐significant (*p* = .06) negative linear selection on VCL, in the sperm limitation treatment (Table 1a). When we included nonlinear terms in the model we identified significant positive correlational selection on PM and flagellum length (Table 1a). Conversely, in the sperm saturation treatment, we found significant negative linear selection on flagellum length, but no quadratic or correlational terms were significant (Table 1b).

**TABLE 1:** The vectors of linear (**β**) selection gradients and matrices (**γ**) of quadratic (diagonals), and correlational (off‐diagonals) selection gradients for sperm traits in the (a) sperm limitation treatment and (b) sperm saturation treatment

(a) Sperm limitation		γ					
	β	BCF	LIN	VCL	PM	HL	FL
BCF	−0.035 ± 0.004	−0.022 ± 0.015					
LIN	0.019 ± 0.005	0.016 ± 0.010	−0.224 ± 0.016				
VCL	*−0.074* ± *0.004*	−0.059 ± 0.008	−0.076 ± 0.007	0.193 ± 0.016			
PM	0.019 ± 0.005	0.088 ± 0.008	0.042 ± 0.007	−0.125 ± 0.011	0.150 ± 0.013		
HL	**−0.209** ± 0.006***	0.013 ± 0.007	0.004 ± 0.006	0.077 ± 0.008	−0.029 ± 0.007	0.001 ± 0.008	
FL	**0.098** ± 0.005**	0.019 ± 0.006	0.005 ± 0.006	−0.015 ± 0.007	**0.18** ± 0.006***	−0.063 ± 0.006	0.013 ± 0.007

(b) Sperm saturation		γ					
	β	BCF	LIN	VCL	PM	HL	FL
BCF	−0.018 ± 0.004	0.220 ± 7.00e−05					
LIN	0.019 ± 0.005	−0.071 ± 2.00e−05	0.025 ± 3.00e−05				
VCL	0.052 ± 0.004	0.076 ± 2.00e−05	0.030 ± 3.00e−05	0.023 ± 5.00e−05			
PM	−0.007 ± 0.006	0.069 ± 3.00e−05	−0.072 ±3.00e−05	0.092 ± 4.00e−05	−0.069 ± 8.00e−05		
HL	−0.052 ± 0.004	−0.009 ± 3.00e−05	0.003 ± 3.00e−05	0.026 ± 4.00e−05	−0.018 ± 5.00e−05	0.008 ± 7.00e−05	
FL	**−0.099** ± 0.005*	0.011 ± 3.00e−05	0.054 ± 3.00e−05	−0.037 ± 4.00e−05	−0.091 ± 5.00e−05	−0.016 ± 4.00e−05	−0.124 ± 7.00e−05

Note

Sperm traits are BCF, beat‐cross frequency; FL, flagellum length; HL, sperm head length; LIN, linearity; PM, percentage of motile sperm; VCL, curvilinear velocity. Gradients are reported ± bootstrapped standard error. Significant gradients are in bold, *p < .05, **p < .01, ***p < .001. Marginally non‐significant terms are italicized.

### 3.3 Canonical rotation analyses

The canonical rotation of the **γ** matrix for the sperm limitation treatment produced two axes of significant nonlinear selection, eigenvectors **m1** and **m5**, which have positive (concave selection) and negative (convex selection) *λ*, respectively (Table 2a). The largest absolute *λ* in this treatment was associated with **m1**. Axis **m1** was also associated with a significant negative *θ* value. Axis **m1** was primarily loaded positively by VCL, and negatively by PM and flagellum length, and axis **m5** was loaded positively by both flagellum length and head length, and negatively by PM (Table 2a). The rotation analysis also identified a significant positive θ on **m3**, which was loaded negatively by BCF and head length (Table 2a). We did not identify any significant eigenvectors in the sperm saturation treatment (Table 2b).

**TABLE 2:** The linear (*θ*) and nonlinear (*λ*) selection gradients for each eigenvector (**m**_i_), produced by canonical rotation of the **γ** matrices in the (a) sperm limitation treatment and (b) sperm saturation treatment

(a) Sperm limitation			Trait loadings (**M**)					
	*θ*	*λ*	BCF	LIN	VCL	PM	HL	FL
**m1**	−0.128**	0.409*	−0.228	−0.120	0.580	−0.652	0.203	−0.362
**m2**	−0.021	0.131	0.018	−0.100	0.741	0.424	0.100	0.501
**m3**	0.203***	0.024	−0.514	−0.080	0.019	−0.195	−0.778	0.294
**m4**	0.048	−0.071	0.813	−0.084	0.198	−0.213	−0.494	−0.060
**m5**	0.007	−0.142*	0.150	0.004	−0.218	−0.555	0.309	0.726
**m6**	−0.022	*−0.240*	−0.001	−0.981	−0.166	0.068	0.072	−0.022

(a) Sperm limitation			Trait loadings (**M**)					
	*θ*	*λ*	BCF	LIN	VCL	PM	HL	FL
**m1**	0.006	0.946	0.835	−0.288	0.320	0.323	−0.016	−0.111
**m2**	0.057	0.065	−0.402	0.111	0.672	0.375	0.351	−0.332
**m3**	−0.013	0.060	0.310	0.773	0.304	−0.297	0.152	0.322
**m4**	0.067	0.004	−0.116	0.229	0.234	0.221	−0.911	−0.025
**m5**	−0.022	−0.140	−0.147	−0.505	0.529	−0.425	−0.089	0.506
**m6**	−0.087	−0.203	−0.096	0.024	−0.139	0.662	0.124	0.719

Note

Significant gradients are in bold, *p < .05, **p < .01, ***p < .001. Original trait loadings for each eigenvector are provided in the **M** matrix. Sperm traits: BCF, beat‐cross frequency; FL, flagellum length; HL, sperm head length; LIN, linearity; PM, percentage of motile sperm; VCL, curvilinear velocity. Marginally non‐significant terms are italicized.

**FIGURE 1:**
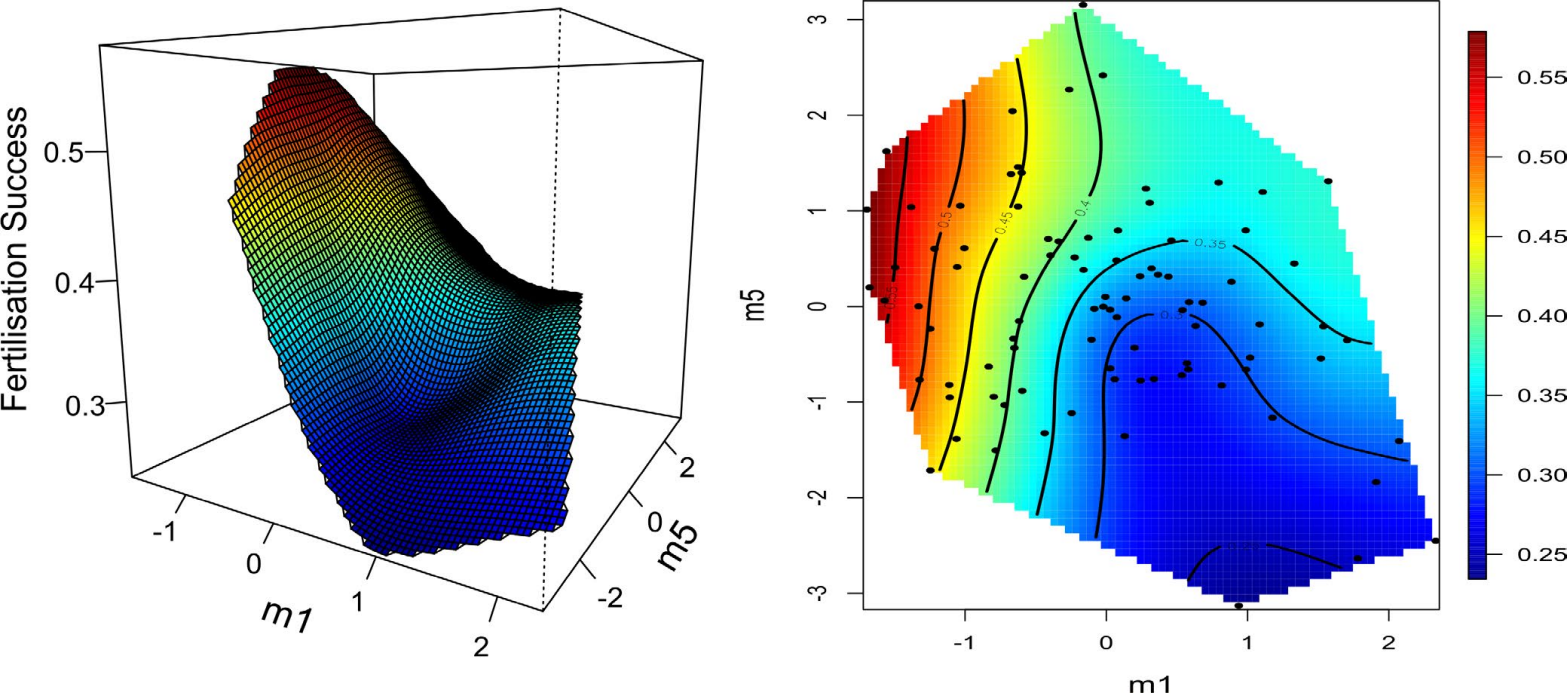
Thin‐plate spline visualization of selection acting on the two major axes of nonlinear selection, **m1** and **m5**, in the sperm limitation treatment. The surface is presented in a three dimensional perspective plot (left panel) and as a contour plot (right panel). Predicted fertilization success is presented on the vertical axis in the perspective plot, and by color in both plots. Red indicates high fitness and blue indicates low fitness. Points on the contour plot represent male scores along these axes. Note: these axes are orthogonal

The fitness surface representing selection on **m1** and **m5** in the sperm limitation treatment depicted negative directional selection on trait combinations along **m1**, with decreasing fitness for positive scores (Figure 1). This indicates that selection favors high scores for PM and flagellum length, and low scores for VCL, that is, a combination of many motile, slow sperm with long flagella. The fitness surface along axis **m5** depicts a peak for intermediate to low scores, though selection appeared relatively weak along this axis (Figure 1). This indicates a slight decrease in fitness for strongly positive and strongly negative scores along **m5**, or for males with divergent combinations of percentage motility and sperm size traits.

### 3.4 Comparison of selection between treatments

The geometric comparisons provided quantitative support for the above qualitative differences in selection gradients from the selection analyses. The *β_i_* gradient vectors from the sperm limitation and sperm saturation treatments were oriented 93.4° from each other, indicating that these vectors are close to orthogonal. The individual *β_i_* gradients were not significantly correlated (*r* = −0.18, *p* = .733). Similarly, the *γ_ij_* gradients in the two treatments were not significantly correlated (*r* = −0.33, *p* = .147), and comparison of the γ matrices returned a Krzanowski score of 1.25 out of 3 (41.7% of score for similarity).

### 3.5 Phenotypic correlation analysis

The Mantel tests revealed that the correlation and partial correlation matrices for the two sets of males did not differ in structure, with significant, strong similarity scores between the two correlation matrices (*r* = 0.93, *p* = .0014), and the partial correlation matrices (*r* = 0.83, *p* = .0042). We therefore present a combined matrix of full and partial correlations for all males (Table 3). We identified five significant partial correlation coefficients in the dataset. These were positive partial correlations between BCF and LIN, LIN and PM, and VCL and PM, and negative partial correlations between LIN and VCL, and PM and FL (Table 3). Finally, the VIFs for each phenotypic trait were all <2 (range = 1.04–1.31).

**TABLE 3:** Phenotypic correlations among sperm traits, with partial correlation coefficients in parentheses

	BCF	LIN	VCL	PM	HL
BCF	–				
LIN	0.390 (0.338)***	–			
VCL	−0.259 (−0.135)	−0.274 (−0.276)***	–		
PM	−0.080 (−0.045)	0.082 (0.224)**	0.410 (0.440)***	–	
HL	−0.104 (−0.074)	−0.088 (−0.087)	0.005 (−0.069)	0.049 (0.062)	–
FL	0.004 (0.008)	−0.074 (−0.095)	−0.054 (−0.054)	−0.086 (−0.039)*	−0.167 (−0.172)

Note

Significant partial correlation coefficients are in bold, *p < .05, **p < .01, *** *p* < .001. Sperm traits: BCF, beat‐cross frequency; FL, flagellum length; HL, sperm head length; LIN, linearity; PM, percentage of motile sperm; VCL, curvilinear velocity.

## 4 DISCUSSION

Our findings confirm that patterns of multivariate selection on sperm motility and morphology traits are dependent on local sperm densities. Using the model broadcast spawning mussel, *M. galloprovincialis*, we found complex patterns of linear and nonlinear selection on sperm phenotypes during sperm limitation, and, in contrast, only linear selection on flagellum length under sperm saturation. A key finding of our study is that sperm morphology was important in both environments, with sperm motility emerging as a key target of selection when sperm were limiting. Furthermore, our fertilization assays were conducted in passive flow conditions that enabled us to assess selection when sperm biology is more likely to impact the outcomes of fertilization (i.e., after gametes have been dispersed by physical properties of the aquatic environment; Crimaldi & Zimmer, 2014). Our finding that sperm motility was targeted by selection during sperm limitation supports the prediction that sperm traits are important mediators of fertilization within the local environment of eggs (Crimaldi & Zimmer, 2014).

The findings reported here add to the accumulating evidence that selection often simultaneously targets multiple components of the sperm phenotype (e.g., Fitzpatrick et al., 2012; Hadlow et al., 2020; Johnson et al., 2013; Lymbery et al., 2018; Monro & Marshall, 2016), and address calls for a better understanding of how changing environmental contexts affect these patterns (Evans & Lymbery, 2020; Liao et al., 2018). Importantly, our study takes the additional step of incorporating a range of functionally integrated motility and morphological traits (Fitzpatrick & Lüpold, 2014; Humphries et al., 2008; Pizzari & Parker, 2009; Simmons & Fitzpatrick, 2012) that are known to influence the dynamics of fertilization under different gamete densities (e.g., Crean & Marshall, 2008). Our results provide empirical support for theoretical predictions that divergent sperm morphologies and swimming ability are favored along the sperm density continuum (Evans & Lymbery, 2020; Levitan, 1998; Lotterhos & Levitan, 2010). Given that variation in local sperm densities is likely to occur both within and between spawning events for external fertilizers, heterogeneous patterns of selection are likely to maintain variation in sperm phenotypes in these taxa.

We found multiple significant axes of selection on sperm trait combinations and a steep selection surface in the sperm limitation treatment, yet no significant axes of selection on trait combinations under sperm saturation. This suggests that lower sperm densities exert more complex selective pressures on sperm phenotypes than higher sperm densities (in the absence of competition from rival males). This finding is perhaps not surprising because chance encounters with eggs are likely to occur more often as sperm density increases, which could reduce the strength of selection on traits that promote sperm‐egg encounters, at least in the absence of rival ejaculates. In contrast, selection under sperm limitation, when chance gamete encounters are less frequent and eggs remain unfertilized for longer, is expected to directly target sperm motility traits. Sperm exhibiting phenotypes that enable them to search efficiently for unfertilized eggs over a longer period of time will achieve greater fertilization success under these conditions (Fitzpatrick et al., 2012; Levitan, 2002). Sperm limitation is therefore expected to select for the conservation of energy while sperm search for unfertilized eggs (Fitzpatrick et al., 2012; Levitan, 2000). Our results were generally consistent with this prediction, showing that selection during sperm limitation generally favored ejaculates comprising many motile, but slower swimming, sperm. This pattern is also consistent with prior work on external fertilizers revealing that slower‐swimming sperm are longer‐lived (Burness et al., 2004; Levitan, 2000) and more efficient at fertilizing eggs under similar conditions imposed in our sperm limitation treatment (Fitzpatrick et al., 2012). Overall, our results highlight the importance of sperm limitation as a selective force shaping sperm phenotypes, particularly sperm motility, in external fertilizers—a finding that is timely given recent suggestions to address drivers of ejaculate and sperm evolution beyond sperm competition (Liao et al., 2018; Parker et al., 2018).

In contrast to the patterns uncovered in the sperm limitation treatment, we found no evidence of nonlinear selection on sperm trait combinations under sperm saturation. Instead, we detected only negative linear selection on flagellum length. When sperm from a single male are overly abundant, polyspermic fertilizations and subsequent embryo death are expected due to high sperm‐egg collision rates. This leads to the somewhat counterintuitive expectation that less “effective” sperm should be favored in sperm saturating scenarios because they are less likely to overwhelm eggs and cause high rates of polyspermy (Levitan, 2018). Our finding that sperm with shorter flagella were favored in the sperm saturating treatment, rather than long flagella as in the sperm limiting treatment, might reflect an advantage for less effective sperm during saturated, monogamous fertilizations. Similar patterns have been shown in studies of urchin gamete compatibility proteins, whereby highly compatible sperm perform well under sperm limitation yet induce polyspermy when sperm are too abundant (Levitan, 2012; Levitan et al., 2019; Levitan & Ferrell, 2006). It is, however, interesting that we did not find evidence of selection on other traits that may mitigate costs of polyspermy when sperm are saturating, such as smaller head sizes (e.g., Johnson et al., 2013). Moreover, our finding that abnormal fertilizations represented only half of the unsuccessful fertilization attempts was lower than expected, since eggs of *M. galloprovincialis* do not have a complete block to polyspermy (Dufresne‐Dubé et al., 1983). It is therefore possible that our population has other adaptations (besides the traits we measured) to prevent high levels of polyspermy. For instance, Crean and Marshall (2008) demonstrated that adult ascidians kept in high density populations produced sperm that induced less polyspermy than those kept in low density populations, although the mechanisms involved remain uncertain. Alternatively, the eggs of this species may be selected to balance the pressures of both sperm abundance and sperm limitation (Levitan, 2004), or may have other adaptations to defend against polyspermy (e.g., Crean & Marshall, 2015; Firman & Simmons, 2013). For example, gamete recognition proteins (Kosman & Levitan, 2014; Levitan & Ferrell, 2006) may differentially mediate gamete interactions along the sperm density continuum in this species. More investigation into the dynamics of sperm‐egg interactions and selection on egg traits in saturating conditions would be useful to test these possibilities.

Our study focused on non‐competitive fertilizations, and therefore the patterns of selection reported here may not be representative of conditions under which ejaculates from different males compete for access to eggs. However, a comparison between the present findings and an earlier study that incorporated competitive fertilizations may begin to disentangle the effects of sperm density and competition on selection. Lymbery et al. (2018) examined multivariate sexual selection on the same suite of sperm traits considered here in *M. galloprovincialis* during competitive fertilizations, although they used sperm densities that were intermediate to the treatments in our current study. There were intriguing differences between patterns of selection in our non‐competitive trials compared to those conducted under conditions of sperm competition. For example, sperm swimming straightness was an important predictor of fertilization success under sperm competition (Lymbery et al., 2018), but not in either of the non‐competitive treatments used here. There could be several explanations for this disparity. For example, selection during competitive fertilizations will target highly efficient fertilizers, as the pressure to outcompete rival males for fertilizations is greater than the pressure to avoid polyspermy—a situation resulting in sexual conflict as females are under selective pressure to reduce polyspermy (Evans & Lymbery, 2020; Levitan, 2012; Levitan & Ferrell, 2006). As discussed above, during monogamous fertilizations selection may favor weaker sperm, as sperm that are too efficient at fertilization will cause polyspermy and embryonic death (Levitan, 2018; Levitan & Ferrell, 2006). Whether selection actually favors weak sperm in nature will depend on the frequency of competitive and non‐competitive fertilizations at high sperm concentrations. We suspect that as *M. galloprovincialis* are often densely aggregated, non‐competitive fertilizations may be less common than competitive fertilizations (Levitan, 2018; Lymbery et al., 2018). However, field experiments that discern the level of sperm competition during natural spawning events are needed to address this question. A second potential explanation for the disparity between the targets of selection in non‐competitive and competitive fertilizations is that the straightness of sperm swimming paths is closely tied to the way sperm search for and track cues from individual eggs (Evans & Sherman, 2013; Kaupp et al., 2006; Riffell et al., 2004), which could be more important in competitive than non‐competitive situations. Finally, the different sperm concentrations employed in both studies may account for the different findings. In order to fully understand and separate the effects of sperm density and competition, it is necessary to repeat the competitive fertilizations at densities comparable to our sperm saturation treatment (Evans & Lymbery, 2020; Liao et al., 2018).

In conclusion, we show that variation in local sperm densities generates distinct patterns of multivariate selection on sperm motility and morphology. These findings indicate that variation in sperm density is a key driver of phenotypic selection on ejaculate traits, particularly when sperm are limiting, and that studying patterns of selection across multiple environments is crucial for understanding the adaptive value of sperm characters. The substantial differences in the shape and targets of selection across different fertilization environments highlights the importance of assessing a broad range of sperm phenotypes (e.g., motility and morphology) when seeking to identify predictors of reproductive fitness. Such divergent patterns of selection likely allow phenotypic variation in sperm traits to be maintained through frequency‐dependent selection (Bell, 2010). We anticipate that the extension of our experimental design to assess patterns of selection under sperm competition at similar densities, and/or different flow regimes, will be valuable for resolving the relative importance of sperm limitation and sperm competition in driving selection on gametes in externally fertilizing systems (Evans & Lymbery, 2020; Liao et al., 2018; Parker et al., 2018).

## CONFLICT OF INTEREST

The authors declare no conflicts of interest.

## AUTHOR CONTRIBUTIONS

**Jessica Hadlow:** Conceptualization (lead); data curation (lead); formal analysis (lead); funding acquisition (equal); investigation (lead); methodology (equal); project administration (lead); visualization (lead); writing – original draft (lead); writing – review and editing (equal). **Rowan Lymbery:** Conceptualization (supporting); data curation (supporting); formal analysis (supporting); investigation (supporting); methodology (supporting); project administration (supporting); supervision (equal); writing – review and editing (equal). **Jonathan P. Evans:** Conceptualization (supporting); data curation (supporting); formal analysis (supporting); funding acquisition (equal); investigation (supporting); methodology (supporting); project administration (supporting); resources (lead); supervision (equal); writing – review and editing (equal).

## Data Availability

Data associated with this manuscript are stored in the Dryad Digital Repository (https://doi.org/10.5061/dryad.z8w9ghxdr).

## APPENDIX A

### ASSESSING THE EFFECT OF BLOCK ID USING SEQUENTIAL SELECTION ANALYSES

To ensure that no single block had a substantial effect on the fit of the models described in the main text, we sequentially removed each of the 22 blocks from the dataset, and for each iteration we re‐ran the selection analyses to estimate linear and nonlinear selection in each treatment (sperm limitation and sperm saturation). The main text provides a more detailed explanation of the selection analyses.

Table A1 provides the range for each of the selection gradient coefficients across the iterations where each block was removed, and the proportion of the iterated models in which each gradient was significant. All models in the sperm limitation treatment revealed significant negative linear selection on head length, and significant negative correlation between PM and flagellum length (Table A1). There was positive linear selection on flagellum length in 77% of the models, and significant linear selection on VCL in 32% of the models (Table A1). This aligns with the analyses presented in the main text, which found significant linear selection gradients for head length and flagellum length, significant correlational selection between PM and flagellum length and marginally nonsignificant negative linear selection on VCL (see Table 1 in main text). In the sperm saturation treatment, 95% of the models yielded significant negative linear selection on flagellum length, which matches the result presented in the main text (Table A1 and Table 1 in main text). None of the 44 ancillary selection analyses described here yielded any significant gradient that was not significant in the initial analyses presented in the main text. We are confident that no single outlier block had a substantial effect on the outcome of the selection analyses, or on our conclusions about the different patterns of selection in sperm limiting and sperm saturating environments.

**TABLE A1:** Range of each linear selection gradient (*β*) and nonlinear selection gradient (*γ*) from models estimating linear and nonlinear selection in the sperm limitation treatment, and in the sperm saturation treatment

	*Sperm Limitation*		*Sperm Saturation*	
	Range	Proportion significant	Range	Proportion significant
*β* gradients				
BCF	−0.056, −0.014	0	−0.043, 0.001	0
LIN	0, 0.043	0	0.006, 0.057	0
VCL	−0.096, −0.053	0.32	0.039, 0.086	0
PM	−0.006, 0.048	0	−0.046, 0.031	0
HL	**−0.241, −0.173**	1.00	−0.077, −0.035	0
FL	**0.081, 0.139**	0.77	**−0.118, −0.053**	0.95
*γ* gradients				
BCF	−0.184, 0.03	0	0.06, 0.29	0
LIN	−0.257, −0.175	0	−0.033, 0.065	0
VCL	0.021, 0.231	0	−0.073, 0.081	0
PM	0.069, 0.178	0	−0.137, 0.138	0
HL	−0.024, 0.039	0	−0.048, 0.047	0
FL	−0.006, 0.2	0	−0.218, −0.085	0
BCF‐LIN	−0.124, −0.012	0	−0.124, 0.053	0
BCF‐VCL	0.006, 0.107	0	−0.083, −0.028	0
BCF‐PM	0.036, 0.174	0	0.072, 0.134	0
BCF‐HL	−0.048, 0.023	0	−0.009, 0.044	0
BCF‐FL	−0.044, 0.041	0	−0.006, 0.056	0
LIN‐VCL	−0.052, 0.067	0	−0.097, −0.065	0
LIN‐PM	−0.169, −0.019	0	0.022, 0.057	0
LIN‐HL	−0.031, 0.03	0	−0.016, 0.027	0
LIN‐FL	0.001, 0.115	0	−0.015, 0.023	0
VCL‐PM	−0.024, 0.19	0	−0.153, −0.014	0
VCL‐HL	−0.007, 0.07	0	0.052, 0.136	0
VCL‐FL	−0.065, 0.006	0	−0.038, 0	0
PM‐HL	−0.042, 0.014	0	−0.079, −0.008	0
PM‐FL	**−0.17, −0.028**	1.00	0.147, 0.207	0
HL‐FL	−0.11, 0.01	0	−0.099, −0.013	0

Note

Within a treatment, each of 22 blocks was sequentially removed from the dataset and the coefficients estimated (i.e., gradient ranges were estimated from 22 models per treatment). Gradients in bold are those that were significant in selection analyses presented in the main text, and the proportion of models in which each gradient was significant is provided. Traits: BCF, beat‐cross frequency; FL, sperm flagellum length; HL, sperm head length; LIN, sperm path linearity; PM, the percentage of motile sperm; VCL, curvilinear velocity.
